# Percutaneous Coronary Intervention in Pregnancy: Modeling of the Fetal Absorbed Dose

**DOI:** 10.1155/2019/8410203

**Published:** 2019-07-07

**Authors:** Kfier Kuba, Diana Wolfe, Alan H. Schoenfeld, Anna E. Bortnick

**Affiliations:** ^1^Department of Obstetrics and Gynecology, Division of Maternal Fetal Medicine, Montefiore Medical Center and Albert Einstein College of Medicine, Bronx, NY, USA; ^2^Department of Obstetrics and Gynecology, Division of Maternal Fetal Medicine, Cardiology Joint Program, Montefiore Medical Center and Albert Einstein College of Medicine, Bronx, NY, USA; ^3^Department of Radiology, Montefiore Medical Center and Albert Einstein College of Medicine, Bronx, NY, USA; ^4^Department of Medicine, Division of Cardiology, Montefiore Medical Center and Albert Einstein College of Medicine, Bronx, NY, USA

## Abstract

There is a gap in the literature regarding fetal radiation exposure from interventional cardiac procedures. With an increasingly large and complex cohort of pregnant cardiac patients, it is necessary to evaluate the safety of invasive cardiac procedures and interventions in this population. Here we present a case of a patient with multiple medical comorbidities and non-ST elevation myocardial infarction (NSTEMI) at 15 weeks' gestation, managed with percutaneous coronary intervention (PCI). We were able to minimize the maternal and estimated fetal absorbed radiation dose to <1 milliGray (mGy), significantly less than the threshold dose for fetal adverse effects at this gestational age.

## 1. Introduction

There are few studies reporting the estimated fetal absorbed dose of invasive cardiac procedures in pregnancy and none reporting it in relation to PCI. Lee et al. reported performing a mitral valvuloplasty in the second trimester for severe rheumatic mitral stenosis with a total radiation exposure equivalent to 1.3mGy [1]. Schrale et al. reported a series of three patients who underwent percutaneous device closure of a patent foramen ovale during pregnancy, each with estimated fetal doses less <0.001mGy [2]. As patients with complex medical comorbidities increasingly experience pregnancy, there is a need for data regarding radiation exposure for PCI as well.

## 2. Case Report

A 28-year-old woman, gravida 4, para 0-0-3-0, called Emergency Medical Services at 15 4/7 weeks of pregnancy complaining of facial and lower extremity edema. In the ambulance, she reported one episode of sharp, substernal chest pain. She had a history of insulin-dependent type 2 diabetes mellitus, hypertension, hypercholesterolemia, nephrotic syndrome with estimated glomerular filtration rate (eGFR) >60mL/min prior to pregnancy, diabetic neuropathy, tobacco use, and poor adherence to medications. Her obstetrical history was significant for three prior first trimester spontaneous abortions in the setting of poorly controlled diabetes, with hemoglobin A1c ranging from 11.5 to 13.9%. Laboratory evaluation for anti-phospholipid syndrome was negative, and evaluation of protein C, protein S, and anti-thrombin III was normal. Her medication regimen included insulin, nifedipine 30 mg extended-release daily, furosemide 80 mg every 8 hours, gabapentin 900 mg every 8 hours, aspirin 81 mg daily, and prenatal vitamins. Prior to pregnancy, she did not present for prepregnancy counseling, had not seen a cardiologist, and was poorly adherent to followup with endocrinology and nephrology services.

**Figure 1:**
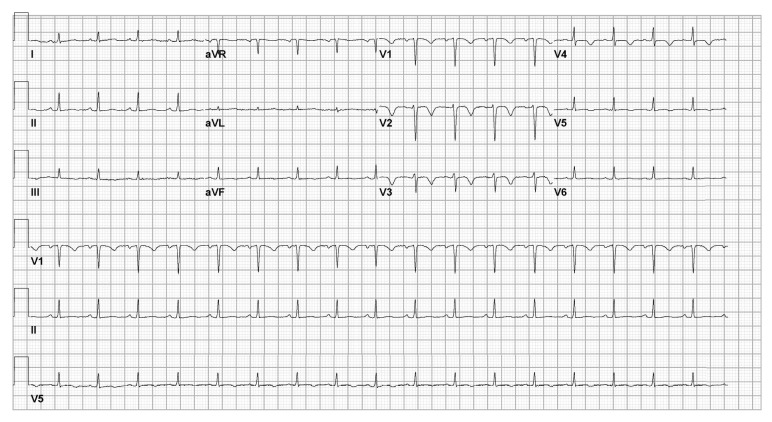
Wellen's T waves in leads V1-V4 consistent with a possible stenosis in the left anterior descending coronary artery.

Upon presentation, the patients' blood pressure was 134/95 mmHg and pulse, 111 bpm, and respirations were 18/min; she was afebrile and oxygen saturation was 100% on room air, with a BMI of 23.6 kg/m^2^. Physical exam revealed anasarca, breath sounds were clear, heart sounds were normal, and bedside ultrasound confirmed the presence of a fetal heart rate. Serum laboratory evaluation revealed newly elevated CPK 614 U/L, troponin T 0.33 ng/mL, pro-BNP 6,312 pg/mL, and eGFR >60mL/min. The patient's electrocardiogram revealed deeply inverted Wellen's T waves in leads V1-V4 (Figure 1). Doppler assessment revealed patency of the lower extremity veins, pulmonary perfusion (Q) scan was not suggestive of pulmonary embolism, and transthoracic echocardiogram revealed no regional wall motion abnormality. Based on objective EKG and cardiac biomarker criteria, she was diagnosed with non-ST elevation myocardial infarction with the likely culprit vessel being the left anterior descending.

**Figure 2:**
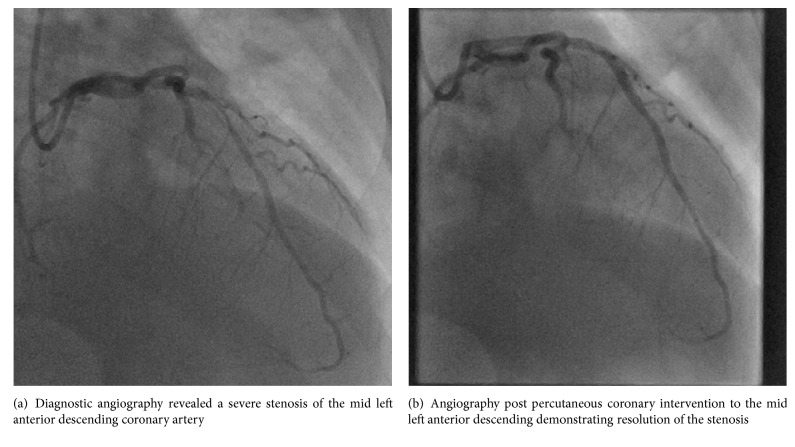


Upon the interdisciplinary recommendation of interventional cardiology, maternal fetal medicine, nephrology, and internal medicine, the patient underwent cardiac catheterization. Left radial approach was used, and a standard cocktail of nitroglycerin, verapamil, and heparin was administered intraoperatively. Catheterization revealed an atherosclerotic 90% stenosis of the mid-left anterior descending artery, which was treated with a single SYNERGY 3x16mm (Boston Scientific, Marlborough, MA, USA) drug-eluting stent (Figures 2(a) and 2(b)). This particular stent was selected as the presence of a bioabsorbable polymer might favor shorter dual antiplatelet therapy. Eliminating clopidogrel after several months could be beneficial should the patient have preterm labor or need cesarean delivery in the context of a high risk pregnancy and multiple comorbidities and because anticoagulation with enoxaparin was to be sustained throughout pregnancy due to hypercoagulability from nephrotic syndrome. It is important to note that spontaneous coronary artery dissection is a common culprit in the pregnant population, and additional intracoronary imaging modalities could have been undertaken to make that diagnosis as well, if the lesion type was not readily apparent. In this case, more diffuse atherosclerosis is noted in the images, supporting the atherosclerotic diagnosis. After the procedure, all aforementioned medications were continued at the same dose, including aspirin 81 mg daily, and the patient was further treated with clopidogrel 75 mg daily, enoxaparin 40 mg daily, metoprolol 25 mg every 12 hours, and pravastatin 20 mg daily, the latter of which was selected based on recent data suggesting favorable safety and pharmacokinetic profiles [3]. As data is limited, use of pravastatin in pregnancy should be individualized and utilized only after careful consideration of the patient-specific risk-benefit ratio.

Radiation exposure was minimized during the case with collimation, low dose fluoroscopy at 15 frames/second, fluoroscopy save for storage of images on a Philips Allura Xper FD10 (Philips, Eindhoven, The Netherlands), and 0.5 mm Xenolite (Lite Tech, Inc. Norristown, PA, USA) was positioned under the abdomen. Iodinated contrast (109 mL) was used. The total air kerma was 311mGy, and the dose-area product was estimated at 20 Gy·cm^2^, over a total of 14.1 minutes of fluoroscopy exposure. The fetal radiation dose was mathematically modeled after the procedure. Total absorbed dose calculations were performed using PCXMC 2.0 software (STUK, Helsinki, Finland), using Monte Carlo methodology [4, 5]. During the PCI, the total absorbed dose at the uterus, a proxy for total fetal absorbed dose, was calculated to be 0.79 mGy.

The hospital course was complicated by acute kidney injury, hyperkalemia, pediculosis capitis, and cystitis. As a result of her complex and worsening medical comorbidities and poor maternal and fetal prognosis, options counseling addressed termination of pregnancy, which the patient declined. Her pregnancy course was later complicated by superimposed preeclampsia, severe fetal growth restriction, and short cervix. At 25 1/7 weeks, the patient went into preterm labor and delivered a 480 g neonate who died at 40 minutes of life from complications of extreme prematurity and intrauterine growth restriction. The patient did not experience a postpartum hemorrhage. In pregnancy, fetal Nuchal Translucency at 11w5d was 1.05mm, and Noninvasive Prenatal Screening results were low risk (<1/10,000) for Trisomies 13, 18, and 21, as well as Monosomy X and triploidy, with fetal fraction of 2.9%. Microdeletion screening for 22q11.2 deletion syndrome was low risk (<1/3,000). No fetal anomalies were seen on routine anatomy ultrasound, and cervical length screening at 19w5d was 2.05cm; however, the patient declined vaginal progesterone.

## 3. Discussion

Optimal management of severe medical comorbidities in pregnancy is best achieved by an interdisciplinary approach. The pregnant cardiac patient is of particular importance as complications of preexisting cardiac disease can predispose to maternal death. Percutaneous coronary intervention (PCI) with revascularization remains the gold standard of therapy for acute coronary syndrome [6]. The decision to recommend PCI can present a challenge to obstetricians and interventional cardiologists who are concerned about radiation risk to the developing fetus. Published data shows that physicians who commonly care for pregnant patients may be unfamiliar with the magnitude of radiation risks in pregnancy. Ratapalan et al. found that 34% of obstetricians surveyed estimated the fetal malformation risk to be 5% or greater with abdominal computed tomography (CT) at 6 weeks' gestation, 5% of whom would recommend pregnancy termination for this indication alone [7]. In fact, CT of the abdomen is associated with an estimated fetal radiation dose of 10-35 mGy, depending on gestational age, body habitus, and exact acquisition parameters, while the estimated threshold dose for induction of major malformations is 200-500 mGy [8].

There is a gap in the literature regarding estimated fetal dose from cardiac catheterization, either diagnostic or percutaneous interventions [1, 2]. With attention to technique, we demonstrate that the estimated fetal dose from PCI can be orders of magnitude lower than CT, <1 mGy in this case. Further dose reduction can be achieved with a lower frame rate (7.5 frames/sec) and use of AlluraClarity (Philips, Eindhoven, The Netherlands) technology which decreases radiation exposure [9]. External shielding of the abdomen and pelvis is a common practice for pregnant patients undergoing catheterization, but interestingly, is likely of limited value. This is because the fetal absorbed dose from fluoroscopy and other chest irradiation procedures is a result of internal Compton scatter from thoracic tissues, rather than direct fetal irradiation from the primary x-ray beam [8, 10]. Objectively useful methods for reducing radiation exposure include collimation of the radiation beam to focus on a smaller area of interest, decreasing frame rate, use of wedge filters, and changing the projection frequently to distribute the radiation rather than concentrate it [11]. Use of a radial artery approach also obviates direct pelvic radiation typically used with femoral artery entry, thereby further reducing the fetal absorbed dose, although this can be circumvented by using ultrasound to localize the vessel.

At higher doses, radiation exposure can be detrimental to the developing fetus. There is an assumed stochastic lifetime cancer risk to the fetus, and these concerns have sparked inquiry into maximizing diagnostic or treatment ability while minimizing medical radiation exposure [12]. However, direct fetal effects are thought to be deterministic, the most clinically significant of which remain pregnancy loss and congenital malformations [8]. The National Council on Radiation Protection and Measurements has reported that the risk of congenital abnormality is negligible at <50 mGy and increased only at doses >150 mGy. More recently, fetal threshold doses of 50 mGy and 200 mGy have been described for conceptus loss (within the first two weeks after fertilization, before implantation) and congenital anomalies, respectively [8]. Current evidence suggests that radiation at doses <100 mGy is not associated with an increase in pregnancy complications or later neurodevelopmental delay [8, 12]. It is important to note that risk from radiation exposure is most pronounced within the first two weeks after fertilization (i.e., before implantation) and continues to lessen as the pregnancy progresses [8, 12].

Per American College of Obstetricians and Gynecologists' guidelines, a pregnant woman should never be denied an indicated procedure solely because she is pregnant, and pregnancy alone is not an absolute contraindication to medical radiation exposure, including PCI [13, 14]. However, the American College of Cardiology has identified radiation exposure from cardiovascular imaging as a concern [11]. This is particularly applicable to the patient presented, as she is likely to undergo many diagnostic imaging studies and interventional procedures over her lifetime. Realistic counseling for the risks and benefits of the proposed diagnostic procedure should be done, with shared decision-making on the part of physician and patient [8, 11, 12].

Consideration should be given to the gestational age, alternative diagnostic modalities, the estimated fetal dose, and in particular, the understanding that the life of the fetus depends upon the life of the mother [10, 12]. The benefits of PCI in this case were considered to outweigh the small risk of radiation exposure to the mother and fetus and attention was given to minimizing maternal and fetal radiation doses. Additionally, medications used in the cardiac catheterization laboratory (nitroglycerin, aspirin, clopidogrel, anticoagulants, calcium channel, or beta blockers) may be used after a patient-specific risk-benefit assessment is made, replacing the traditionally used pregnancy risk categories (e.g., A, B, C, D, X) used for medications [15].

The patient presented in her second trimester, a time at which risk of forming new congenital anomalies is negligible, and risk of fetal neurodevelopmental delay is not expected below a threshold dose of 60mGy [14]. Consideration was given to performing diagnostic CT coronary angiography rather than PCI, but it was ultimately decided against given the contrast-associated risk of nephrotoxicity and time lost in obtaining the study, which, if positive, would necessitate followup PCI, resulting in treatment delay and overall higher radiation exposure.

It is unlikely that radiation exposure, including her PCI and pulmonary perfusion scan, had a meaningful contribution to preterm delivery as the maternal comorbidities were known risk factors for preeclampsia, growth restriction, preterm birth, and poor neonatal outcome. Nevertheless, utilizing the techniques described allowed minimization of the modeled fetal absorbed dose of ionized radiation.

## 4. Conclusion

The interventional team was able to perform successful PCI at an estimated fetal absorbed dose of <1mGy, well below guideline recommended threshold dose for significant fetal effects. Prospectively modeling and reporting estimated fetal radiation doses from pregnant women undergoing cardiac catheterization procedures should be encouraged in order to better understand the burden and outcomes of in-utero exposure in this cohort.

## References

[1] Lee M. M., Sohn D. W., Oh B. H. (1992). Percutaneous mitral valvuloplasty in a mid-term pregnant woman with severe rheumatic mitral stenosis. *The Korean Journal of Internal Medicine*.

[2] Schrale R. G., Ormerod J., Ormerod O. J. (2007). Percutaneous device closure of the patent foramen ovale during pregnancy. *Catheterization and Cardiovascular Interventions*.

[3] Costantine M. M., Cleary K., Hebert M. F. (2016). Safety and pharmacokinetics of pravastatin used for the prevention of preeclampsia in high-risk pregnant women: A pilot randomized controlled trial. *American Journal of Obstetrics & Gynecology*.

[4] Metzger R. L., Van Riper K. A. (1999). Fetal dose assessment from invasive special procedures by Monte Carlo methods. *Medical Physics*.

[5] Dauer L. T., Thornton R., Boylan D. C. (2011). Organ and effective dose estimates for patients undergoing hepatic arterial embolization for treatment of liver malignancy. *Medical Physics*.

[6] Bob-Manuel T., Ifedili I., Reed G., Ibebuogu U. N., Khouzam R. N. (2017). Non-ST elevation acute coronary syndromes: a comprehensive review. *Current Problems in Cardiology*.

[7] Ratnapalan S., Bona N., Chandra K., Koren G. (2004). Physicians' perceptions of teratogenic risk associated with radiography and CT during early pregnancy. *American Journal of Roentgenology*.

[8] Dauer L. T., Thornton R. H., Miller D. L. (2012). Radiation management for interventions using fluoroscopic or computed tomographic guidance during pregnancy: a joint guideline of the society of interventional radiology and the cardiovascular and interventional radiological society of europe with endorsement by the canadian interventional radiology association. *Journal of Vascular and Interventional Radiology*.

[9] Abuzeid W. (1878). *Radiation safety in the cardiac catheterization lab: A time series quality improvement initiative*.

[10] Colletti P. M., Lee K. H., Elkayam U. (2013). Cardiovascular imaging of the pregnant patient. *American Journal of Roentgenology*.

[11] Bortnick A. (2017). *Shared Decision-Making in Radiation Exposure for Patients and Operators: An Interventional Perspective*.

[12] Wagner L. K. American college of radiology practice guideline for imaging pregnant or potentially pregnant adolescents and women with ionizing radiation.

[13] ACOG (2017). ACOG committee opinion no. 696: nonobstetric surgery during pregnancy. *Obstetrics & Gynecology*.

[14] ACOG (2017). ACOG Committee Opinion No.723: Guidelines for diagnostic imaging during pregnancy. *Obstetrics & Gynecology*.

[15] Lal R. Drugs in Pregnancy and Lactation: Improved Benefit-Risk Information. https://www.fda.gov/downloads/drugs/developmentapprovalprocess/smallbusinessassistance/ucm431132.pdf.

